# StaBle: Staggered PRF With DouBle Transmission for Increasing the Velocity Limit of High-Frame-Rate Vector Doppler Imaging

**DOI:** 10.1109/ojuffc.2026.3664322

**Published:** 2025-02-12

**Authors:** GERALDI WAHYULAKSANA, COLIN K. L. PHOON, GLENN I. FISHMAN, JEFFREY A. KETTERLING

**Affiliations:** 1 Department of Radiology, Weill Cornell Medicine, New York, NY 10022 USA; 2 Division of Pediatric Cardiology, Hassenfeld Children’s Hospital at NYU Langone Health, New York, NY 10016 USA; 3 Leon H. Charney Division of Cardiology, NYU Grossman School of Medicine, New York, NY 10016 USA

**Keywords:** Dealiasing, echocardiography, intracardiac blood flow, transmission scheme, vector Doppler imaging

## Abstract

Vector Doppler Imaging (VDI) addresses the limitations of traditional Doppler imaging by measuring blood flow in axial and lateral directions but will produce incorrect results if aliasing is present. Aliasing becomes more likely when using high transmit frequencies such as in small animal cardiac applications. The use of multiple transmit angles decreases the Nyquist limit, which further increases the likelihood of aliasing. A new transmission scheme, termed StaBle, is proposed to increase the Nyquist limit of conventional sequential angle VDI by multiple fold. StaBle combines the velocity limit extension of staggered multiple pulse repetition frequency (PRF) with a double transmission scheme. With three transmit angles and two PRFs, StaBle was able to achieve a 6–12 times higher velocity limit compared to sequential angle VDI. Simulation and phantom spinning disk experiments were conducted to evaluate StaBle’s performance. The simulation results showed a normalized root-mean-squared error of less than 5% compared to an ideal vector field in both axial and lateral directions. Phantom results showed a 9-fold improvement in detecting peak axial velocity over sequential three angle VDI. The ability of StaBle to obtain an unaliased vector field *in vivo* was demonstrated by imaging a mouse left ventricle where the Doppler signal was corrupted by aliasing artifacts using just a double transmit scheme. The resolved estimated vector velocity showed consistent beat-to-beat variation in velocity, confirming StaBle’s robustness under realistic conditions and its potential for use in investigative studies.

## I. INTRODUCTION

DOPPLER ultrasound is one of the most used imaging modalities to study cardiovascular hemodynamics but it is only sensitive to the axial velocity component of flow. Although blood flow is typically unidirectional, complex vortex flow patterns, which feature axial and lateral flow components, occur at various locations within the cardiovascular system [1]. These vortices, if they can be visualized and quantified, can provide insights into heart conditions [2], [3]. Ultrasound vector flow imaging is a technique that allows visualization of the axial and lateral flow components. It can be performed with various approaches, such as particle image velocimetry (echo-PIV) [4], multibeam Doppler [5], transverse oscillations [6], and color-Doppler-based vector-flow mapping [7]. However, the implementation of these methods with sequential data acquisition in standard ultrasound scanners limits the region of flow for which temporal and spatial resolution is maintained [8]. The advancement of ultrafast ultrasound imaging overcomes these limitations in resolution [8]. Multi-angle plane-wave vector Doppler imaging (VDI) achieves fine temporal resolution, is robust to out-of-plane flow, and does not require ultrasound contrast agent to achieve sufficient sensitivity [9], [10]. While ultrafast imaging reduces the decorrelation artifacts present in larger flow areas of sequential systems, aliasing remains a challenge in cardiac plane-wave Doppler imaging.

In Doppler imaging, the maximum detectable velocity is constrained by the Nyquist velocity limit ($V_{N}$) of pulse-Doppler ultrasound is defined by:

(1) $$V_{N}=\frac{PRF\lambda }{4},$$

where λ is the wavelength of the transmit center frequency and PRF is the pulse repetition frequency between two consecutive pulses. For a typical cardiac probe with a center frequency of 3 MHz and a PRF of 5 kHz (15 cm depth range), the Nyquist limit is 64 cm/s. Imaging high blood flow velocities in the aorta (with a mean peak velocity of 1.2 m/s) [11] or the E wave in mitral regurgitation patients (≥ 1.5 m/s) [12] presents challenges. While plane-wave Doppler imaging offers enhanced temporal resolution across the entire field of view, the Nyquist velocity limit is reduced compared to conventional Doppler imaging methods for two reasons. First, the Nyquist PRF is reduced relative to the absolute transmit PRF by the number of transmit angles (M) [8], [10]. Second, Nyquist PRF is reduced because the round-trip time for plane-wave beamforming is based on the distance from the edge of the transducer to the far corner of the image rather than the maximum axial image depth.

Aliasing is also a concern in small animal models, such as mice, which are widely employed to investigate cardiovascular dynamics because they are easy to manipulate genetically and possess many similarities to humans [13], [14], [15]. Implementing VDI in mouse models poses challenges because mouse hearts are an order of magnitude smaller than human hearts [16]. As a result, high-frequency ultrasound (HFU) (≥ 15 MHz) is required to achieve adequate spatial resolution [17]. Although finer spatial resolution is achieved by increasing the transmit center frequency (eq. 1), the increased frequency lowers the Nyquist velocity limit, which increases the likelihood of aliasing artifacts at peak flow velocities.

To provide context for murine cardiac imaging, the aliasing threshold is 12.3 cm/s for multi-angle VDI using a 31-MHz linear array with an axial imaging depth of 10 mm (15 mm diagonal), three transmit angles, and an absolute PRF of 30 kHz. The transmitral E-wave velocity in a baseline mouse model ranges from 54–81 cm/s [18], [19], [20], whereas in the aorta, the peak velocity may surpass 1 m/s [21], [22]. Therefore, there is a need to increase the velocity limit significantly beyond the Nyquist limit to mitigate the issues of aliasing in VDI of murine cardiac blood flow.

Several methods have been proposed to extend the Nyquist velocity limit in ultrasound Doppler. One direct method is by unwrapping the phase of the aliased signal [23]. Dealiasing has been implemented on individual Doppler shifts [9] or using least-squares estimation to solve for multiple angles [24]. An unsupervised learning [25] and deep learning approach was adopted for phase unwrapping [26], [27]. However, these methods are limited to addressing 1- or 2-fold aliasing, and the robustness of the unsupervised learning approach in VDI for handling various angles has not been demonstrated. An alternate method is the extended least-squares vector Doppler method by Ekroll et al. [28], where the least squares regression method and block matching were combined to extend the Nyquist limit by a factor of 5. This method is less effective in the presence of low signal-to-noise ratio and high spatial velocity gradients which are frequently encountered in cardiac imaging [28].

Another approach is to repeat transmits of each plane-wave angle [29] (e.g., [1,1,2,2...]) rather than using the common sequential angle transmission (e.g., [1,2,1,2...]). This strategy improves the velocity limit by the number of transmission angles relative to the sequential method. Typically, 2 to 5 transmit angles are utilized in VDI [10], which results in a 2 to 5-fold increase in the Nyquist limit when using double transmit. Using our previous example of a 31-MHz probe with 3 transmission angles, the double transmit method would increase the Nyquist velocity limit to 36.6 cm/s. However, this is still below the required velocity range for murine cardiac blood flow.

A dealiasing method that uses a staggered PRF has been successfully implemented in radar signal processing [30], [31]. Dealiasing was performed in post-processing by comparing the phase wrapped velocities that occur with each PRF. This approach, with a dual-PRF scheme, was successfully translated to color Doppler ultrasound imaging to extend the Nyquist limit by a factor of *p* (ranging from 2–4 times) [32]. However, implementation with a conventional VDI transmission sequence in cardiac imaging remains a challenge because it requires the true flow velocity to remain nearly constant during the transmit pattern. However, flow varies rapidly over a cardiac cycle. In practice, the long, multi-angle transmission sequence of VDI, especially when using a triple PRF to achieve a 5-fold increase in the Nyquist limit, makes the translation of staggered PRF to cardiac applications difficult. The dual-wavelength approach is conceptually similar to the staggered PRF method, but only extends the Nyquist limit up to a factor of 2 [33].

In this paper, we overcome the above challenges by implementing a new transmission scheme termed StaBle (Staggered PRF with douBle Transmission). StaBle extends the velocity aliasing limit of the commonly used sequential angle VDI transmission by multiple fold. StaBle combines a staggered PRF with double angle transmission, resulting in a velocity extension that is the product of the two techniques. We provide a brief overview of the methods that StaBle is built upon (vector Doppler estimation, staggered PRF, and double transmission) and then describe how to implement StaBle. We validate the approach with simulation and phantom experiments using a spinning disk phantom. We then demonstrate the method *in vivo* with a mouse cardiac model.

## II. THEORY AND PROCESSING IMPLEMENTATION

### A. MULTI-ANGLE VECTOR DOPPLER ESTIMATION WITH LEAST-SQUARES REGRESSION

Multi-angle VDI extends conventional Doppler (i.e., line-based Doppler ensembles) by using velocity estimates from multiple transmit angles to calculate the axial and lateral velocity, and has the effect of mitigating the strong transmit angle dependency typical in conventional Doppler [9]. The method is implemented by transmitting M plane waves at transmit angles Tx, $\left(\theta_{1},\theta_{2},\ldots ,\theta_{M}\right)$ and deriving N virtual receive angles Rx, ($\phi_{1},\phi_{2},\ldots ,\phi_{N}$) for each Tx. A 3-Tx, 3-Rx approach is a good compromise between accuracy and temporal resolution [10]. Therefore, we adopted M=3 for our implementation. Velocity estimates, $U_{mn}$, were obtained for each Tx-Rx pair (M×N) using the lag-one autocorrelation technique [34] as is done for conventional Doppler imaging.

(2) $$U_{mn}=PRF_{eff}\frac{c_{o}}{4\pi f_{0}}arg\left\{R_{mn}(1)\right\},$$

(3) $$\text{for}R_{mn}(l)=\frac{1}{P-1}\sum_{i=0}^{P-2}x_{mn}^{*}(i)x_{mn}(i+l),$$

where $PRF_{\mathrm{eff}}$ is the effective PRF between the transmission pulses with the same Tx angle $\left(PRF_{\mathrm{eff}}=PRF/M\right)$ for sequential angle transmit sequence), $c_{o}$ is the speed of sound, $f_{0}$ is the transmit center frequency, $R_{mn}$ is the autocorrelation value (l=1 for lag-one), and $x_{mn}$ is the slow time signal of window length P. We can use a trigonometry identity to define the relation of $U_{mn}$ to the axial, $v_{z}$, and lateral, $v_{x}$, velocity

(4) $$v_{z}\left(cos\;\theta_{m}+cos\;\phi_{n}\right)+v_{x}\left(sin\;\theta_{m}+sin\;\phi_{n}\right)=2U_{mn}.$$

Matrix A is defined based on the Tx and Rx pairs and constructs a linear system to obtain $v_{z}$ and $v_{x}$ based on the Doppler velocity u:

(5) $$\left[\begin{array}{cc} cos\;\theta_{1}+cos\;\phi_{1} & sin\;\theta_{1}+sin\;\phi_{1}\\ \vdots & \vdots \\ cos\;\theta_{M}+cos\;\phi_{N} & sin\;\theta_{M}+sin\;\phi_{N} \end{array}\right]\left[\begin{array}{l} v_{z}\\ v_{x} \end{array}\right]=2\left[\begin{array}{c} u_{11}\\ \vdots \\ u_{MN} \end{array}\right]$$

which is solved with least squares regression to find v:

(6) $$v=\left[\begin{array}{c} v_{z}\\ v_{x} \end{array}\right]={\left(A^{T}A\right)}^{-1}A^{T}u.$$

### B. STAGGERED PRF

The unambiguous estimated Doppler velocity is limited by the Nyquist velocity limit ($V_{N}$), defined by eq. 1. However, when aliasing occurs, the measured Doppler velocity ($U_{mn}$) is not the true velocity but can be defined as

(7) $$U_{mn}=V_{D}^{u}-2n_{N}V_{N},$$

where $V_{D}^{u}$ is the unambiguous (non-aliased) Doppler velocity, and $n_{N}$ is the Nyquist number or aliasing order (in the case of no aliasing, $n_{N}=0$). Because the aliasing order is always an integer, we can rearrange (7) to

(8) $$n_{N}=\text{floor}\left(\frac{V_{D}^{u}\;+\;V_{N}}{2V_{N}}\right)\text{.}$$

The actual Doppler velocity can be inferred if the Nyquist number is known. However, in actual measurements, the aliasing order is unknown. A staggered PRF transmission can be utilized to estimate the aliasing order, thereby extending the Nyquist velocity limit and obtaining the actual velocity. This is accomplished by transmitting another sequence of pulses, $PRF_{i}$, with a PRF less than the PRF of the main pulse sequence, $PRF_{1}$ (i.e., $PRF_{i}<PRF_{1},i>1$). Because $V_{N}$ has a linear relation to PRF,

(9) $$PRF_{i}=\frac{p_{i}}{q_{i}}PRF_{1}\Rightarrow V_{Ni}=\frac{p_{i}}{q_{i}}V_{N1}$$

where $p_{i}$ and $q_{i}$ are positive integers, $p_{i}<q_{i}$, and their greatest common divisor is 1. If aliasing is present, the perceived velocity ($U_{mn}$) will vary with different PRFs. By observing the different possible perceived aliased shifts at different PRFs, we can infer a common aliasing coefficient ($n_{\mathrm{coeff}}$) and determine the true velocity.

(10) $$n_{\mathrm{coeff}}=round\left(q_{i}\frac{U_{{mn}_{i}}\;-\;U_{{mn}_{1}}}{2V_{N_{1}}}\right)$$

where round() means rounding to the nearest integer. Based on $n_{\mathrm{coeff}}$, we can obtain $n_{N1}$ and $n_{Ni}$ using a lookup table (an example can be found in Posada et al. [32]) and calculate the unambiguous Doppler velocity using a weighted mean

(11) $$V_{D}^{u}=\frac{\sum_{i}\frac{q_{i}}{p_{i}}V_{D}^{u}}{\sum_{i}\frac{q_{i}}{p_{i}}}=\frac{\sum_{i}\frac{q_{i}}{p_{i}}\left(V_{D_{i}}\;+\;2n_{N_{i}}V_{N_{i}}\right)}{\sum_{i}\frac{q_{i}}{p_{i}}}.$$

**FIGURE 1.**
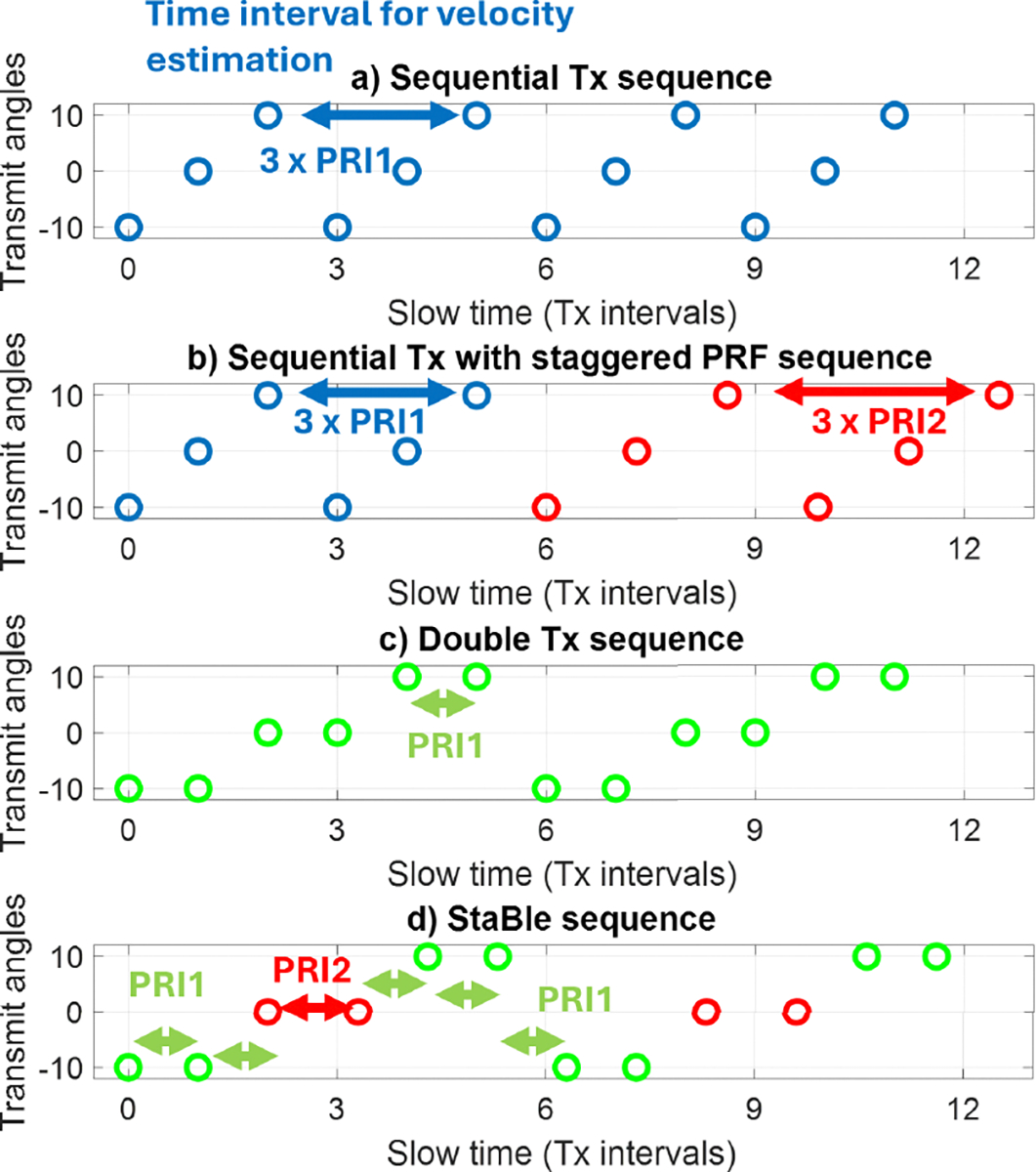
Multi-angle Doppler transmission sequences. a) Sequential angle transmission, b) sequential angle transmission with staggered PRF, c) double transmission, and d) the proposed StaBle transmission scheme.

The Nyquist velocity limit increases based on the selected $p_{i}$ of the PRF ratio. For instance, if $PRF_{2}=3/4PRF_{1}$, it will result in a three-fold increase in Nyquist velocity. However, increasing the ratio also leads to higher errors. In practice, two staggered PRFs are reliable up to $p_{i}=5$ [32]. Therefore, achieving an improvement of more than five-fold requires the use of three staggered PRFs. However, implementing VDI with three or more staggered PRFs and a conventional transmission sequence is not ideal in cardiac blood flow applications. The dealiasing operation assumes the estimated velocity between the multiple PRFs ensemble to be the same, which is violated if there is high (de-)acceleration of blood flow velocity. The use of three Tx angles and two staggered PRFs (Fig. 1b) is already at a disadvantage in estimating pulsating cardiac blood flow because it assumes the velocity remains constant for the moving average window length of *P* × 12-pulse duration instead of 6 pulses in the conventional Tx sequence (Fig. 1a). When using three staggered PRFs, it is even more unlikely that the blood flow velocity is constant for the duration of *P* × 18 pulses. Therefore, extending the Nyquist limit by more than five times with staggered PRF and 3-angle VDI using a conventional transmit sequence is unreliable in practice where blood flow velocity can change within 1 ms.

### C. DOUBLE TRANSMIT SEQUENCE

The conventional plane-wave transmission sequence involves transmitting the M angles sequentially (Fig. 1a) such that the beamformed Rx data can be coherently compounded to generate a high-resolution image. This approach minimizes the compounding time interval to M-1 times the pulse repetition interval (PRI=1/PRF) but is not ideal for VDI because the time interval between the same Tx angle to perform the lag-one autocorrelation of each angle is also M×PRI. As a result, the Nyquist velocity limit relative to the PRI is reduced by the number of transmit angles, where $PRF_{\mathrm{eff}}=PRF/M$.

To address this limitation, Podkowa et al. [29], proposed a double transmit sequence where the same angle is transmitted repeatedly instead of sequentially (Fig. 1c). Using this approach, the lag-one autocorrelation can be performed over a single PRI period, effectively increasing the Nyquist velocity limit by a factor of M compared to the conventional transmission scheme. The autocorrelation is now calculated from

(12) $$R_{mn}(l)=\frac{1}{P/2\;-\;1}\sum_{i=0}^{P/2-2}x_{mn}^{*}(2i)x_{mn}(2i+l).$$

However, the double-transmit sequence has two drawbacks. First, as shown in eq. 12, the Doppler ensemble length is halved over a fixed gate time because the lag-one autocorrelation can only be performed for the pair (every 2 time points) of transmissions with the minimum transmit times. Second, the resulting high-resolution image is not optimal due to the increased time span of the compounding (2M−1)×PRI. For cardiac blood flow imaging, the benefits of increasing the velocity limit outweigh these two drawbacks. Moreover, the use of the double transmit sequence enables the optimal use of the staggered PRF method.

### D. STAGGERED PRF WITH DOUBLE TRANSMISSION (STABLE)

As noted, extending the Nyquist velocity limit with existing techniques such as extended least-squares [28] and staggered PRF [32] by more than 5 times is impractical. Here, we propose the StaBle transmission scheme that combines transmitting double transmission pulses with staggered PRF on different TX angles pairs (Fig. 1d). This approach accumulates velocity extension from both methods and enables an increase in the velocity limit by more than 6-fold. For example, with M=3, double transmission extends the velocity limit by 3 times and, when multiplied by the p of the PRF ratio (2)-(4), results in 6–12 times velocity extension compared to the sequential angle VDI method (Fig. 1d). Another advantage of the StaBle sequence is that it only requires half the number of pulses to perform the dealiasing process compared to the sequential angle with staggered PRF sequence (Fig. 1b). This is particularly important when dealing with pulsating blood flow, where a large number of pulses could lead to inaccuracies due to potentially measuring different velocities.

Post-processing steps were applied to extend the velocity measurements beyond the traditional VDI Nyquist limit. First, a wall filter was applied to the full stack of radiofrequency (RF) data acquired for each transmission angle. Second, the filtered RF data were used to beamform low-resolution images for each Rx steering angle. Third, lag-one autocorrelation was applied to the slow-time signals of each Tx-Rx pair using (12) and Doppler velocity, $\left(U_{mn}\right)$, was estimated using (2). Because double transmission was utilized, the estimated velocity had a limit M=3 times higher than the conventional VDI transmission scheme. Finally, the staggered PRF method was used to unfold any velocity aliasing. The values of $n_{\mathrm{coeff}}$ were calculated with (10) using the staggered PRF pairs between $Tx_{1}\;\&\;Tx_{2}$ and $Tx_{2}\;\&\;Tx_{3}$.

Care must be taken to ensure the staggered PRF dealiasing process is performed with matched net steering angles θ+ϕ. A non-zero net steering angle results in a rotation of the point spread function (PSF) that could lead to velocity estimate errors. In our configuration, the largest total net steering angle of 15° occurs for the combinations $Tx_{1}Rx_{1}$ and $Tx_{3}Rx_{3}$. However, the default receive angles of (−7.5, 0, 7.5°) and transmit angle of 0° for $Tx_{2}$ do not allow for a net matching of $|\theta +\phi |=15^{\circ }$. To obtain net angle symmetry for $PRF_{2}$, we introduce the additional receive steering angles $\phi_{4}=-15^{\circ }$ and $\phi_{5}=15^{\circ }$. Table 1 summarizes the Tx-Rx angle combinations that result in matched net steering angles for staggered-PRF-based dealiasing.

**FIGURE 2.**
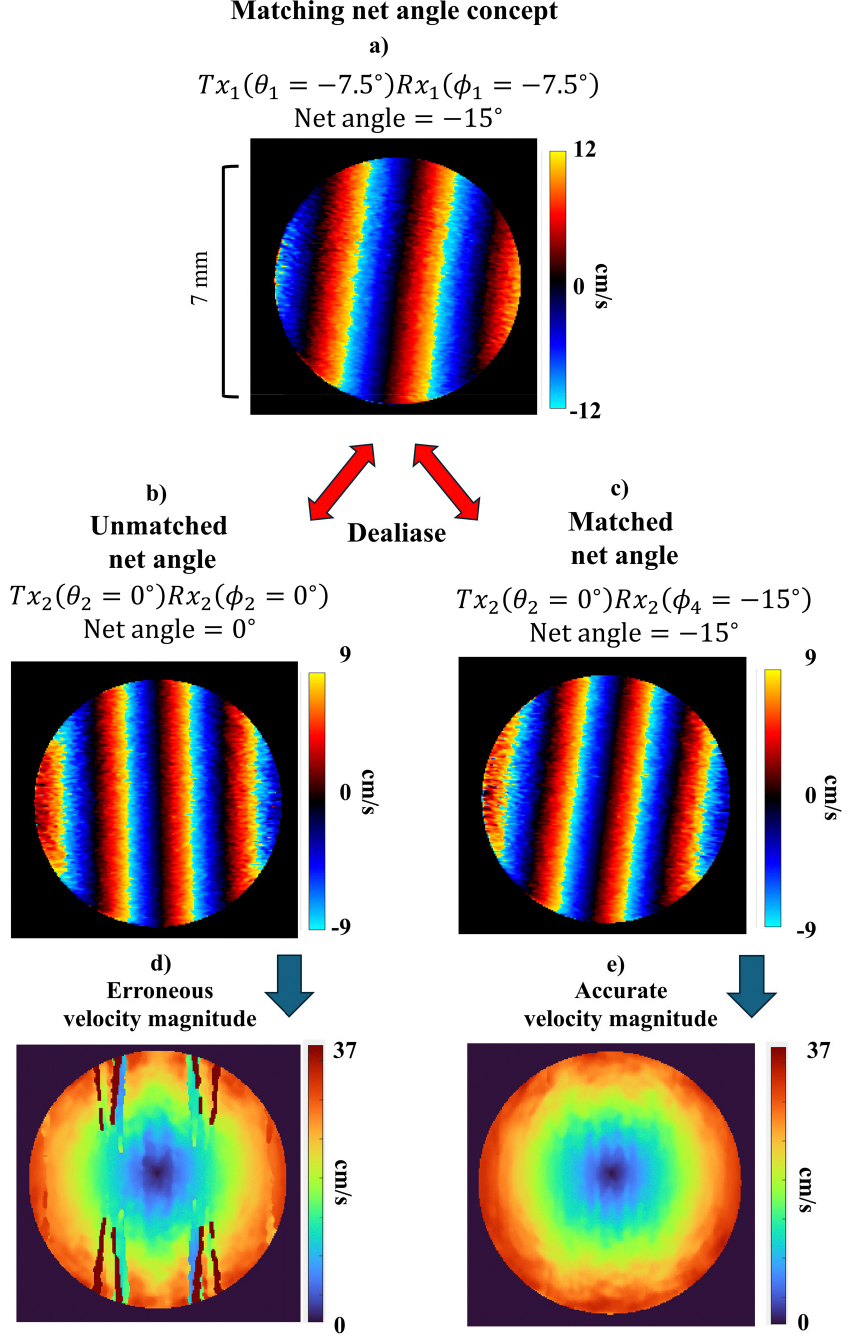
Depiction of dealiasing performed on unmatched net angle (a and b) and matched net angle (a and c). The velocity magnitude estimated from d) the unmatched net angle was erroneous, whereas the estimate from e) the matched net angle was more accurate.

The effect of matched versus unmatched net angles is illustrated in Fig. 2. When $n_{\mathrm{coeff}}$ was computed using unmatched net angles (Figs. 2a and b), the resulting velocity estimates were erroneous (Fig. 2d). In contrast, the calculation on Tx–Rx pairs with identical net steering angles for ${\mathrm{PRF}}_{1}$ (Fig. 2a) and ${\mathrm{PRF}}_{2}$ (Fig. 2c) resulted in accurate velocity estimates (Fig. 2e).

**FIGURE 3.**
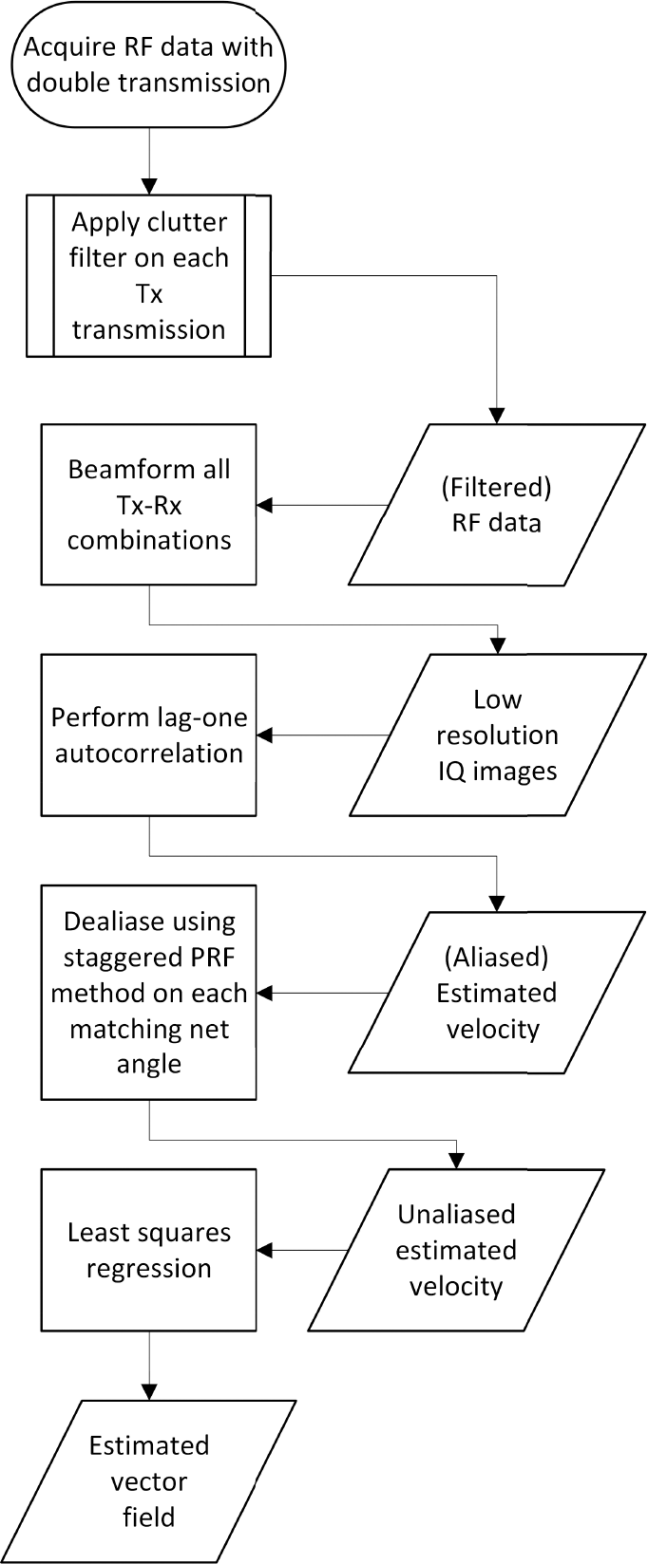
Flowchart of the key processing steps to implement StaBle algorithm.

After calculating $n_{\mathrm{coeff}}$ for all Tx-Rx angles, the Nyquist number $n_{N}$ can be obtained by using a lookup table derived from the selected PRF ratio [32]. The non-aliased Doppler velocity ($U_{mn}$) was obtained using (7). Once all pixels at each time step have been dealiased, the final vector Doppler estimates were obtained using the least-squares regression method as described in Section II-A. The steps to implement StaBle algorithm are illustrated by Fig. 3.

## III. EXPERIMENTAL METHODS

### A. SPINNING CYLINDER

We first evaluated the performance of StaBle using a simulation of a rotating cylinder phantom. We then performed bench-top experiments with a tissue mimicking rotating cylinder phantom. The cylinder was rotated at a constant angular velocity and with an angular acceleration to test the ability of StaBle to resolve flow that is accelerating and decelerating flow.

#### 1) ULTRASOUND PLANE WAVE SEQUENCES

High-frame-rate plane-wave data were acquired with a Vantage 256 (Verasonics, Redmond, WA) and a 256 element, 30–40 MHz linear array (MS550D, FUJIFILM VisualSonics, Inc, Toronto, ON, Canada). Beamforming was performed with MATLAB (MathWorks, Natick, MA) on a 0.5*λ* grid using GPU processing.

#### 2) SIMULATION

**FIGURE 4.**
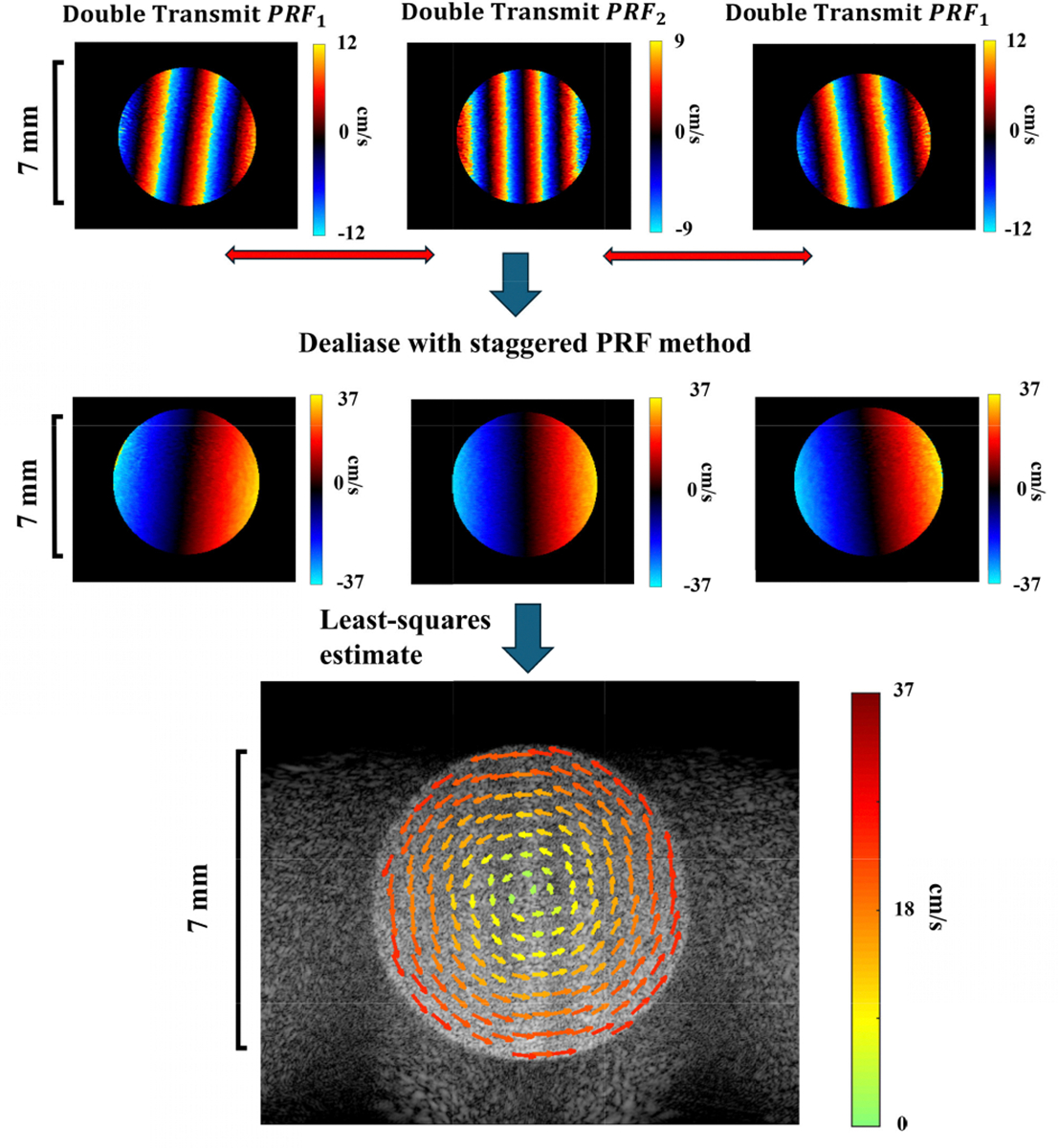
Example of the data processing steps of StaBle. The first row displays heavily aliased color Doppler images (saturated colormap) from individual double transmits at ${\mathrm{PRF}}_{1}$ and ${\mathrm{PRF}}_{2}$. The second row presents the resolved color Doppler after performing the dealiasing process on each Tx-Rx pair with matching net steering angles. Not every pair is shown in the figure. The third row shows the vector field obtained from the resolved color Doppler using the least-squares estimate.

The simulation data were generated using the Verasonics simulation mode with the transmission settings in Table 2. First, we simulated a 3.5 mm radius disk that rotated with a constant angular velocity where the maximum velocity at the edge of the disk was 95% of each (*p*/*q*) ratio extended velocity limit. An example of StaBle data processing and the resulting unambiguous vector field are shown in Fig. 4. We then simulated the same disk but rotated with a velocity profile that had a peak acceleration of 4000 cm/s^2^ and a peak velocity 4.4 times the minimum velocity. The maximum velocity at the edge of disk was still kept at 95% of the extended velocity limit (*p*/*q* = 2/3). The PRF for the constant velocity disk simulation was set to match the phantom experiment whereas the PRF for the varying velocity simulation was adjusted to correspond to the *in vivo* experiment that had blood flow with varying velocities.

To evaluate the accuracy of the StaBle velocity estimates, we generated an ideal reference vector field. The absolute error was calculated using the rootmean square error (RMSE), defined as $RMSE\;=\sqrt{\frac{1}{n}\sum_{i=1}^{n}{\left(y_{i}-{\overset{ˆ}{y}}_{i}\right)}^{2}}$, where y denotes the velocity of each pixel within the disk. The relative error was determined using the normalized RMSE, which is defined as $\mathrm{NRMSE}=\text{RMSE}/(y_{\max}-y_{\min})$, with the range (max- min) referring to the ideal vector velocity. We added white Gaussian noise so that the RF data achieved a signal-to-noise ratio of 40 dB.

#### 3) EXPERIMENTAL PHANTOM

An ≈ 0.5-cm diameter cylindrical tissue-mimicking phantom (ATS, Bridgeport, CT) containing scattering particles was rotated with a DC-servo motor. The phantom was rotated at ≈ 11.5 revs/s, which resulted in a velocity of 18.7 cm/s at the outer edge of the phantom. The probe was clamped to a holder and held in a fixed position relative to the phantom. We acquired data using a conventional sequence, a double transmit sequence, a double transmit sequence with 3x higher PRF, and StaBle (*p*/*q* = 3/4).

### B. IN VIVO MOUSE MODEL

We evaluated StaBle performance *in vivo* in a mouse cardiac model. We scanned the left ventricle (LV) and obtained the vector velocity estimates of the blood flow inside the chamber.

The mouse study was performed following the guidelines set by the Association for the Assessment and Accreditation of Laboratory Animal Care with protocols approved by the New York University School of Medicine Animal Care and Use Committee. The study included male adult mice that were 50 weeks old. The mice were given anesthesia using a low-flow digital anesthesia machine (SomnoSuite, Kent Scientific, Torrington, CT) with 2.5% isoflurane for induction and 1% isoflurane for maintenance, mixed with medical oxygen at a flow rate of 1 L/min. Chest hair was removed to improve acoustic coupling. Core temperature, respiration rate and ECG were monitored.

For the mouse experiments, we used a Vantage NXT 256 (Verasonics) and MS400 probe (Fujifilm VisualSonics) to acquire high-frame-rate plane-wave data using the transmission settings in Table 3. The acquisition was not fully continuous in time because the acquisition rate exceeded the data transfer bandwidth. Consequently, there was an intermittent pause to transfer data between inquisition blocks. One acquisition block contained ≈ 2 cardiac cycles. Clutter filtering was performed using a high-pass filter with a 5% fractional bandwidth cutoff. The filter was applied separately to the even and odd components of the time-domain signal in accordance with the polyphase decomposition theorem, which allows equivalent filtering across interleaved uniform sub-samplings [35]. The LV was manually segmented to remove velocity estimates from areas outside the chamber.

## IV. RESULTS

### A. SIMULATION

**FIGURE 5.**
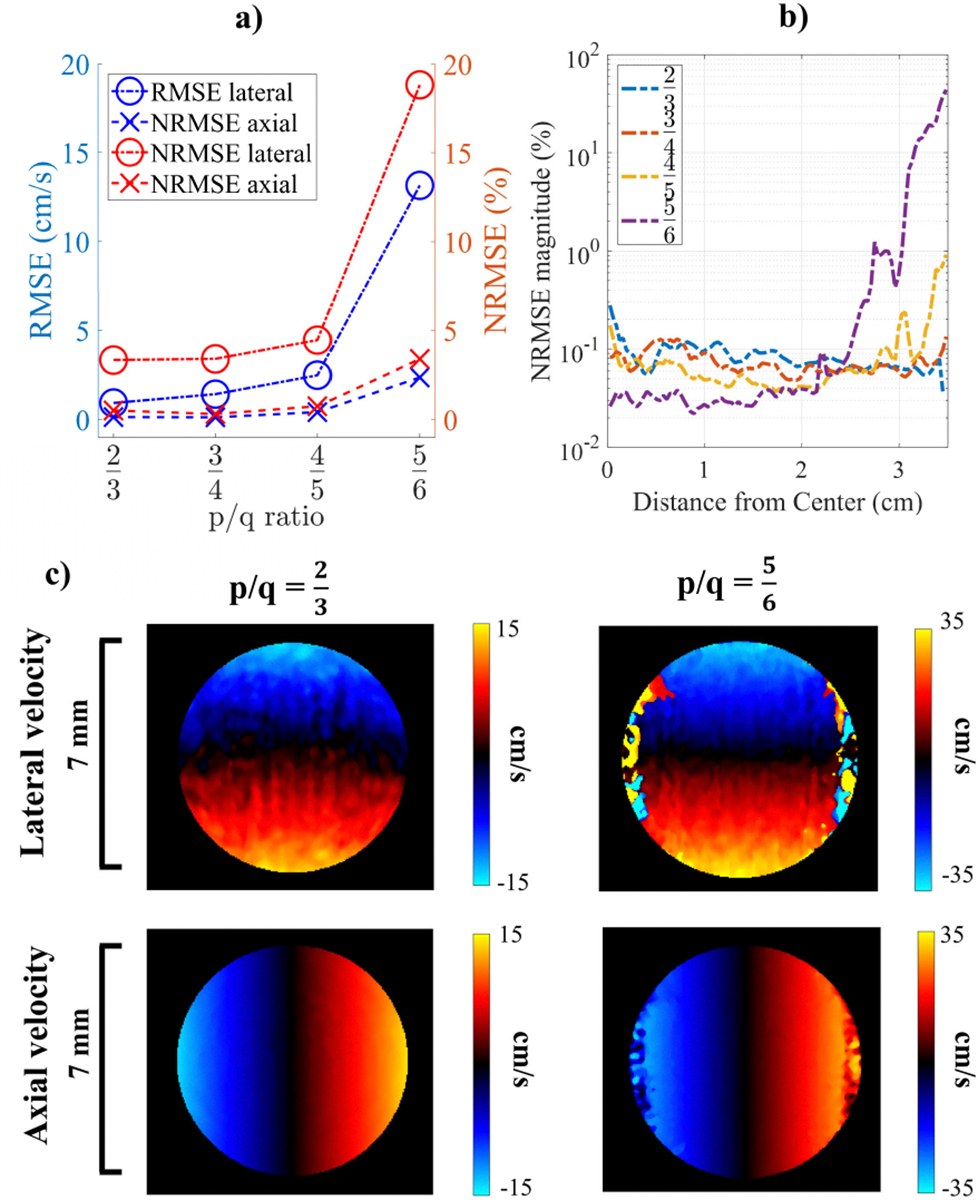
Comparison of the simulated vector field produced by StaBle with varying *p*/*q* ratios and the corresponding ideal vector field. a) Global RMSE (blue) and NRMSE (red). b) The velocity magnitude NRMSE as a function of distance from the center. c) The lateral and axial velocity for *p*/*q* = 2/3 and *p*/*q* = 5/6, with the disks spinning at 95% of their respective maximum extended velocity limit.

The performance of StaBle for increasing *p*/*q* ratios in comparison to their respective ideal vector field is shown in Fig. 5. Axial velocity displayed a lower error than lateral velocity for every *p*/*q* ratio. This is expected because Doppler velocity estimates are most sensitive to axial motion. The RMSE and NRMSE values rose with increased velocity extension (*p*). For *p* ≤ 4, the NRMSE for axial and lateral measurements remained below 5%. However, the dealiasing performance deteriorated drastically at *p* = 5, with axial and lateral NRMSE increasing to 13 and 19%, respectively (Fig. 5a).

The velocity magnitude obtained by StaBle at different *p*/*q* ratios, compared to the ideal vector field, is shown in Fig. 5b. The RMSE near the center of the disk (below 1.75 cm radius) remained below 3% for all *p*/*q* ratios and represented the accuracy of the velocity estimates when there there was no aliasing. For *p* < 4, the NRMSE across all distances stayed nearly constant (<3%), whereas for *p* ≥ 4, the NRMSE increased with distance from the center, reaching 9% for *p* = 4 and 44% for *p* = 5 at the disk’s edge.

**FIGURE 6.**
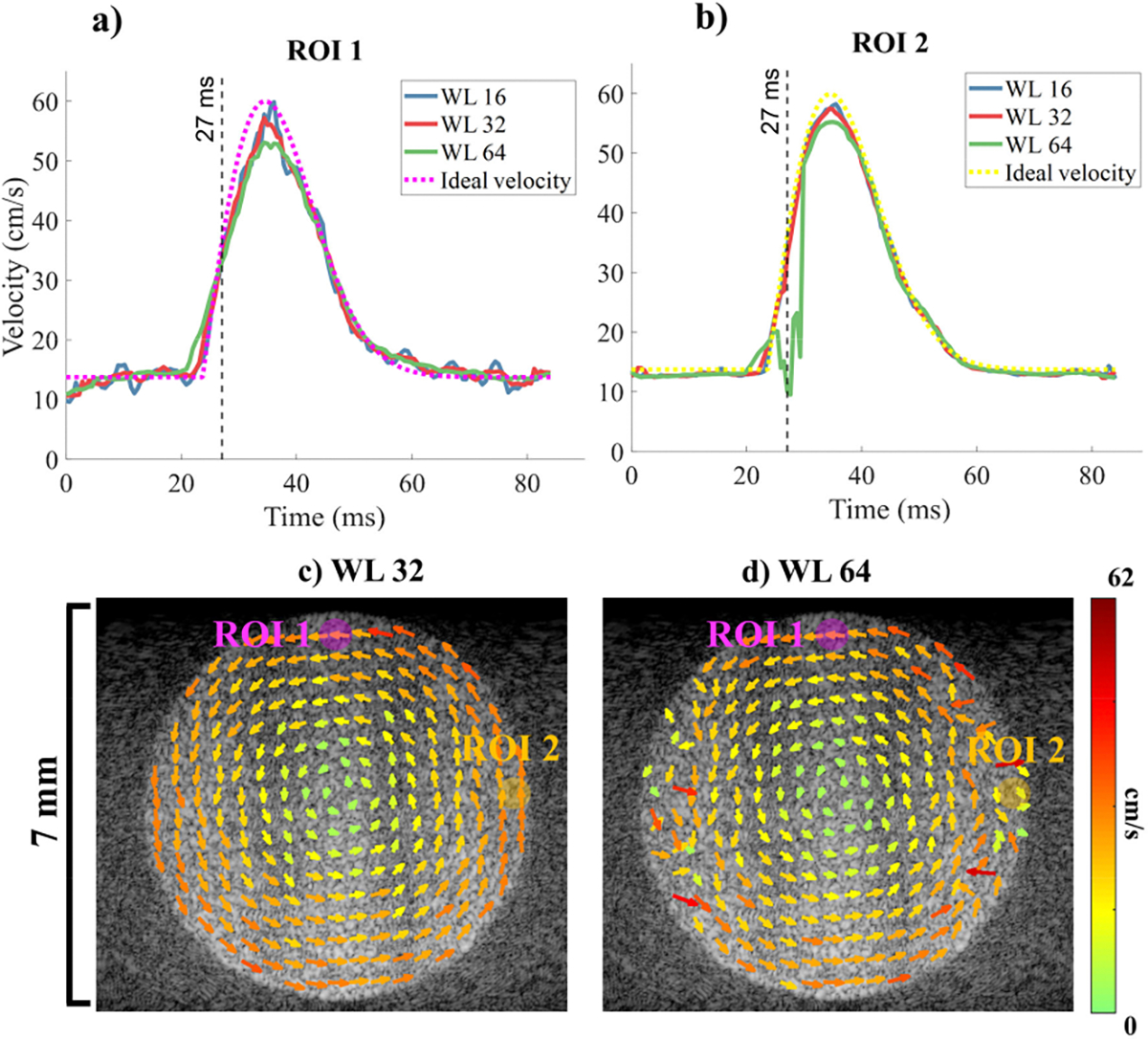
Simulation of a rotating disk with varying speeds. a) The calculated velocity and the ideal velocity profile analyzed by StaBle using different average window lengths for a) lateral velocity in ROI 1 (magenta in c&d) and b) axial velocity in ROI 2 (yellow in c&d). VDI calculated by StaBle during rapid acceleration at 27 ms, processed with window lengths of c) 32 and d) 64.

The ability of StaBle to deal with acceleration and deceleration was evaluated with various average window lengths (Fig. 6). For all window lengths, the estimated velocity at the edges (ROI 2), where velocity was primarily axial, showed a closer alignment with the ideal velocity than the estimated velocities at the top (ROI 1), where velocity was mainly lateral. Window lengths of 16, 32, and 64 samples provided similar NRMSE values, with lateral measurements at 8.4, 7.5, and 9%, respectively. However, the axial measurements with a window length of 64 were too long to accurately capture the acceleration, resulting in a poor NRMSE of 5.5%. Around 38 ms, the axial velocity estimation (Fig. 6b) for the 64-length window showed significant variability. In contrast, window lengths of 16 and 32 showed accurate axial velocity estimates with NRMSE values of 1.7 and 1.8%, respectively.

### B. EXPERIMENTAL PHANTOM

**FIGURE 7.**
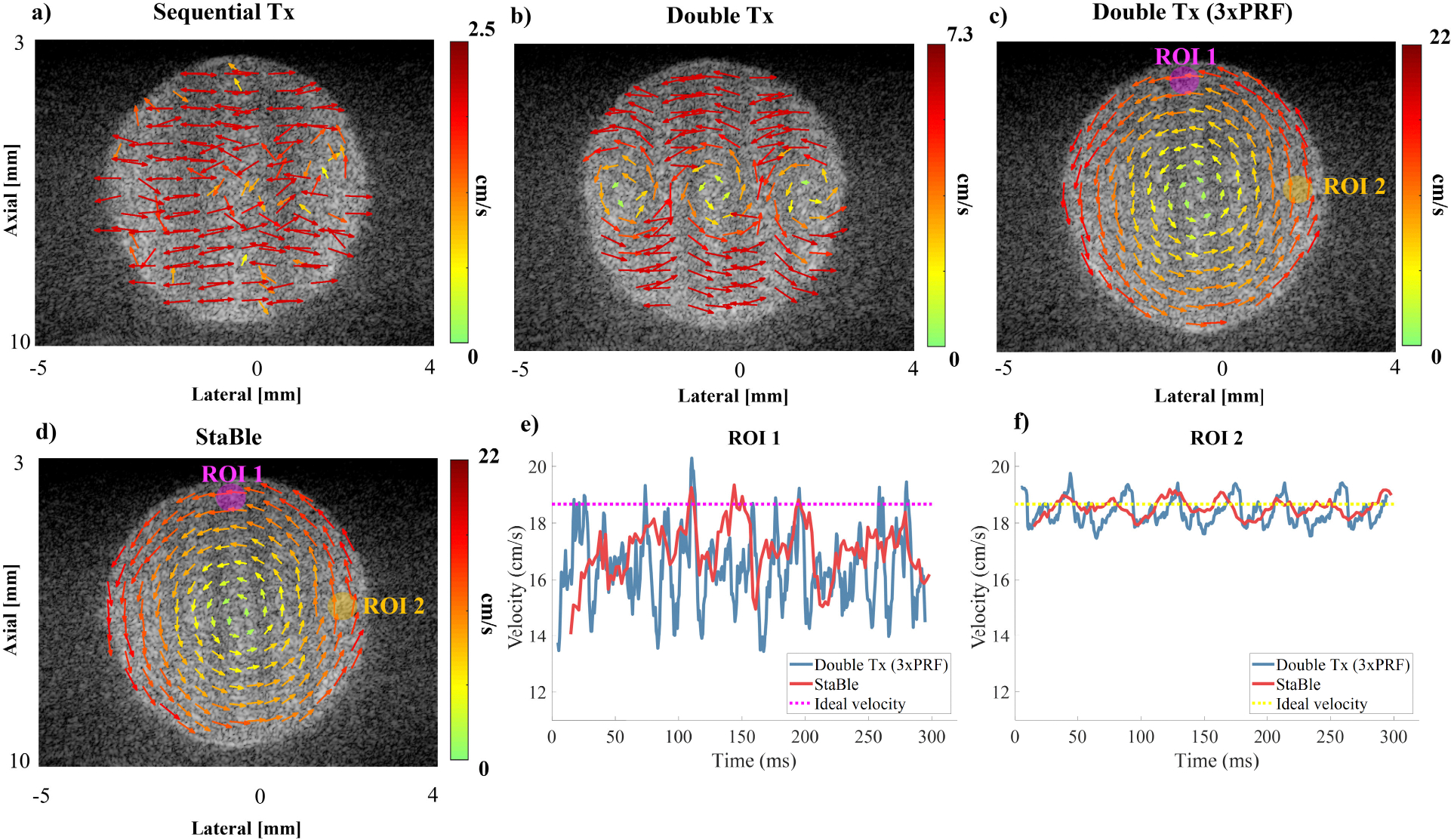
Examples of VDI obtained from the phantom experiment using various transmission sequences. a) Sequential angle transmission sequence, b) Double Tx, c) double transmission with 3x higher PRF, and d) StaBle. The magenta and yellow circles indicate the ROI for obtaining the velocity profile in the lateral (e) and axial (f) directions, respectively.

VDI of the spinning disk using different transmission sequences is depicted in Fig. 7. The rotation was maintained at a constant rate for all transmission sequences (11.5 rev/s, maximum velocity at edge of 18.67 cm/s). The sequential angle sequence failed to accurately represent the rotation due to the disk’s speed being nearly 9x faster than its velocity limit (Fig. 7a). The Double Tx sequence revealed some improvement compared to the conventional sequence but still contained aliasing artifacts. This was due to the disk still spinning 3x faster than its velocity limit (Fig. 7b). The double transmission at 3x higher PRF (Fig. 7c) and StaBle (Fig. 7d) successfully measured the velocity and direction of the rotation when the axial velocity on the outer edge was 85% of the velocity limit. The mean lateral velocity measured by Double Tx (3x PRF) and StaBle was 16.27 ±1.5 cm/s and 16.51 ±2 cm/s, respectively (Fig. 7e). The mean axial velocity measured by Double Tx (3x PRF) and StaBle were 18.41 ±0.46 cm/s and 18.48 ±0.37 cm/s, respectively (Fig. 7f).

### C. MOUSE LEFT VENTRICLE

**FIGURE 8.**
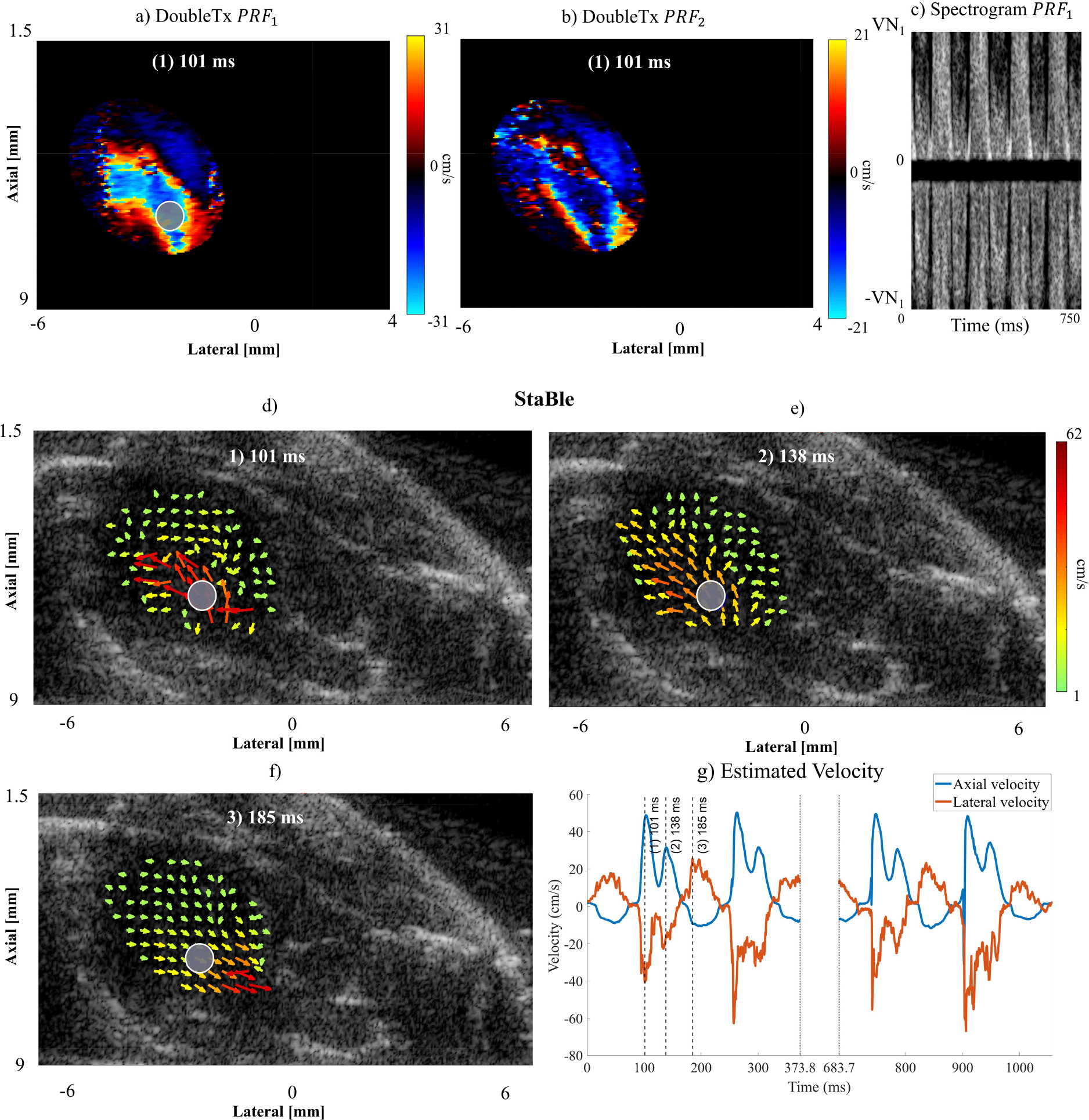
Mouse left ventricle using different transmission sequences. The top row shows aliasing artifact for color Doppler with double transmission at a) ${\mathrm{PRF}}_{1}$ and b) ${\mathrm{PRF}}_{2}$. c) A spectrogram from circle region in ${\mathrm{PRF}}_{1}$ (a). The unambiguous VDI obtained using StaBle over a cardiac cycle at d) early diastole, e) mid-diastole, and f) systole. The gray circles highlight the region of interest used for vector field quantification. g) The axial and lateral velocity derived from the vector field produced by StaBle. The gap between the two central black dashed lines signifies a discontinuity in the acquisition.

Fig. 8 shows the implementation of StaBle to address velocity aliasing in a mouse LV and the resulting intracardiac flow quantification. Aliasing was observed in the color Doppler of Double Tx ${\mathrm{PRF}}_{1}$ and ${\mathrm{PRF}}_{2}$ (Fig. 8a and b), despite having a velocity limit 3x higher than the sequential transmit sequence with the same respective PRF. Fig. 8c shows the aliasing in the pulsed wave Doppler spectra from the color Doppler sample. The staggered PRF dealiasing approach was then applied to resolve the aliasing. Subsequently, all VDI images were generated using the least-squares regression method applied to the unambiguous color Doppler images. The resulting resolved VDI on different cardiac phases is shown on Fig. 8 d-f. Axial and lateral velocities were derived from the resolved vector field to assess blood flow in the LV. The estimated velocities were consistent from beat to beat, showing robust performance of StaBle in resolving velocity aliasing. However, due to rapid acceleration, there were some inaccuracies in the estimated axial velocity of the incoming jet flow during diastole in the third and fourth cardiac cycles (see Fig. 8g). A video depicting the resolved VDI is available as a supplementary material.

## V. DISCUSSION

We introduced a novel transmission scheme (StaBle) that integrates the Double transmission and staggered PRF techniques to significantly increase the velocity limit of the commonly used sequential angle VDI. The double transmission minimizes the autocorrelation time interval at a given PRF, while the staggered PRF extends the velocity range beyond its Nyquist limit. The total improvement, relative to sequential-angle plane-wave Doppler, is represented by the product of the number of transmission angles (*M*) and the numerator of the staggered PRF ratio (*p*). For our experiments, we selected three transmission angles, which resulted in a velocity improvement of 6–12x compared to the sequential angle approach. We used the sequential angle Nyquist limit as our reference because it is the commonly used method [8], [10], [28], [33]. Three angles were chosen because they provide a major improvement compared to two transmission angles, whereas increasing to five transmission angles only provides minor improvement [10]. Additionally, we have introduced the concept of aligning the net steering angle to address errors when comparing staggered PRF with different transmit steering angles for dealiasing. The multi-fold velocity limit extension is especially beneficial for high-frequency cardiac imaging with rapid blood flow. There is a potential to extend StaBle by incorporating three PRFs for three or more transmit angles to accommodate lower PRF or higher transmit frequency but the effectiveness of dealiasing in these schemes remains to be evaluated.

We assessed StaBle’s performance under controlled conditions by conducting simulation and phantom studies using a spinning disk. For the simulation study with a constant velocity, we compared the vector field resolved by StaBle to an ideal vector field and found that StaBle performed well with an NRMSE below 5% in the axial and lateral directions when using a parameter *p* ≤ 4, resulting in up to 12x velocity improvement. StaBle also performed consistently (*p* ≤ 4) across the entire velocity range of the disk (Fig. 5). This indicates that we can utilize StaBle even in the absence of aliasing. The simulation of the spinning disk with a varying velocity demonstrated that StaBle is capable of estimating velocity during acceleration and deceleration. However, the length of the averaging window is a crucial aspect (Fig. 6); a window length of 64 was excessively long and led to estimation inaccuracies in the axial direction.

In the phantom experiment, we evaluated the vector fields produced by sequential angle, double Tx, and double Tx with 3x higher PRF, alongside the StaBle transmission method (*p*/*q* = 3/4), The outer edge of the spinning disk rotated at a velocity 9x greater than the Nyquist limit of the sequential angle method. The results showed that StaBle provided mean and standard deviation velocity estimates comparable to those obtained with the double transmission method at 3x higher PRF. We also performed an *in vivo* study of LV blood flow in a mouse. We observed that the selected transmission settings (31 MHz and ${\mathrm{PRF}}_{1}=25\;\text{kHz}$) with the Double transmission approach led to aliasing which resulted in inaccurate and unreliable flow quantification. However, the aliasing issue was resolved with StaBle, allowing for improved flow quantification.

To analyze the unaliased vector field obtained from StaBle, we estimated the axial and lateral velocities. We observed several erroneous velocity estimates in certain areas during the diastolic inflow jet, leading to fluctuations in the measured velocity. Because this only happens during the jetting period, it was likely due to the window length being too long relative to the high acceleration and was similar to what we observed in the simulation with varying velocity and a window length of 64 frames. We evaluated a window length of 8 and 16 frames, but the error still occurred. Given that the acceleration rate for the disk simulation was comparable to the LV data and we were able to resolve the velocity with window lengths of 16 and 32 frames, the errors in the *in vivo* data were likely caused by a combination of high acceleration and inadequate signal quality.

We conducted simulation, phantom and *in vivo* experiments, but faced some challenges with the spinning disk phantom and *in vivo* experiments. The spinning disk phantom, which had a diameter of 5 mm and was not completely rigid, became increasingly unstable and rotated off-axis at higher rotation rates. As a result, we could not achieve a velocity that maintained a stable rotation with a desired aliasing level when the PRF was set near the theoretical limit of 30 kHz. Therefore, we used 11.5 revolutions per second, where the rotation remained relatively stable, and adjusted the PRF to achieve the desired aliasing level. Doppler methods are relative in the sense that a reduction in real velocity means PRF can be scaled down by the same amount. Even after lowering the rotation speed, we encountered off-axis instability, indicated by the periodic fluctuations noted in the Double Tx and Stable methods. For the phantom experiments, we include an additional case with a 3x higher PRF to avoid bias caused by experimental errors. However, we did not calculate the root mean square error (RMSE) over the entire disk (Fig. 7).

The limitations in achievable experimental rotation rates required the use of different PRFs for the phantom and *in vivo* experiments, and the simulation PRFs were adjusted accordingly. The constant velocity simulations used PRFs from phantom experiments, whereas the varying velocity simulations adopted *in vivo* PRFs. For the *in vivo* experiments, we did not acquire unaliased sequential-angle VDI or double transmission cases because the necessary PRF was not attainable. The peak velocity magnitude that we obtained ≈ 60cm/s falls within the reported *in vivo* range of 54–81 cm/s and the values across different cardiac cycles were consistent. Our methodology shows promise for establishing ground truth at a high frequency transmission (≥ 30MHz).

Moreover, StaBle is effective despite variations in experimental parameters, illustrating its applicability across different PRFs. We also encountered limitations with the ultrasound scanner system, specifically where the data transfer bandwidth led to interruptions in our acquisition sequence, resulting in intermittent pause between different cardiac cycles.

StaBle effectively removed velocity aliasing in our experiments, although with some limitations. First, double transmit generated only half the number of Doppler ensembles compared to a conventional transmit scheme due to uneven sampling rates. Second, the staggered PRF technique only provided the final velocity estimation and was unable to produce a corrected Doppler spectrum such as with pulsed wave Doppler (Fig. 8c). Nevertheless, this is not a major concern because flow quantification can still be obtained from the resulting vector field, and a color Doppler image can still be generated. Third, matching the net-steering angle (transmit and receive) involved beamforming images of the additional receive angles ($\phi_{4}$ and $\phi_{5}$), which increased computation time because the dealiasing calculation needed to be performed on every pixel of each pair of transmit and receive angles. Furthermore, these additional receive angles must be twice that of the transmitting angles to achieve the desired net matching angle. This imposed a constraint to maintain a low transmit angle, preventing the (doubled) receiving angle from being too large and introduced a receive-angle grating lobe artifact. Finally, StaBle will fail if the blood-flow acceleration is too high, because it is still limited by the assumption that the velocity remains nearly constant within a specific set of transmit angles. This implies that the ideal average window length is influenced by the rate of acceleration; longer windows will result in more robust estimates, particularly under challenging conditions such as cardiac imaging.

Clutter filtering is an important factor in Doppler imaging. Although we only used a conventional frequency-based filter to simplify the data processing, other clutter filters may be more suitable. We did evaluate an alternative clutter filter by processing the *in vivo* data with the widely used spatiotemporal singular value decomposition (SVD) method [36]. SVD filtering was performed over each block of uninterrupted acquisition data (half of the total duration) with automatic selection of the rank based on maximizing spatial correlation [37]. SVD filtering was applied to the RF data for each transmission angle prior to the beamforming process. We found that the estimated dealiased velocity results were comparable to the more basic high-pass filtering that we used to demonstrate StaBle. Thus, these results demonstrate that StaBle is not dependent on the choice of clutter-filter.

Despite the use of low transmit frequencies (2)-5 MHz) in human cardiac imaging, aliasing remains a prevalent issue in Doppler imaging due to high peak flow velocities and the low PRF needed for deep penetration (up to 20 cm). Implementing StaBle in human cardiac applications would require some modifications, such as using a phased array probe with diverging wave transmissions, which tend to degrade signal quality, particularly near the image edges. However, Posada et al. [32] showed that the staggered PRF dealiasing method for color Doppler was effective in human cases with a phased array probe.

## VI. CONCLUSION

HFU VDI is advantageous for small-animal imaging due to its ability to provide fine spatial resolution of blood flow. However, as the transmit frequency and number of transmit angles increase, aliasing becomes more likely as the Nyquist limit decreases. We developed a new transmission scheme, StaBle, that increased the Nyquist limit by multiple fold compared to the commonly used sequential angle VDI transmission scheme. Our simulation, phantom, and *in vivo* experiments demonstrate that StaBle performed well across all scenarios. StaBle proved to be an effective tool to obtain unambiguous blood flow velocity, particularly in the challenging case of the mouse LV.

## Supplementary Material

supp1-3664322

This article has supplementary downloadable material available at https://doi.org/10.1109/OJUFFC.2026.3664322, provided by the authors.

## Figures and Tables

**TABLE 1.** Matched net angle dealiasing configuration with 3 transmit angles (−7.5°, 0°, and 7.5°) and 2 PRFs

Net angle	$PRF_{1}$	$PRF_{2}$

−15°	$Tx_{1}\left(\theta_{1=-7.5^{\circ }}\right)\;Rx_{1}\left(\phi_{1=-7.5^{\circ }}\right)$	$Tx_{2}\left(\theta_{2=0^{\circ }}\right)\;Rx_{4}\left(\phi_{4=-15^{\circ }}\right)$
−7.5°	$Tx_{1}\left(\theta_{1=-7.5^{\circ }}\right)\;Rx_{2}\left(\phi_{2=0^{\circ }}\right)$	$Tx_{2}\left(\theta_{2=0^{\circ }}\right)\;Rx_{1}\left(\phi_{1=-7.5^{\circ }}\right)$
0°	$Tx_{1}\left(\theta_{1=-7.5^{\circ }}\right)\;Rx_{3}\left(\phi_{3=7.5^{\circ }}\right)$	$Tx_{2}\left(\theta_{2=0^{\circ }}\right)\;Rx_{2}\left(\phi_{2=0^{\circ }}\right)$
0°	$Tx_{3}\left(\theta_{3=7.5^{\circ }}\right)\;Rx_{1}\left(\phi_{1=-7.5^{\circ }}\right)$	$Tx_{2}\left(\theta_{2=0^{\circ }}\right)\;Rx_{1}\left(\phi_{1=-7.5^{\circ }}\right)$
7.5°	$Tx_{3}\left(\theta_{3=7.5^{\circ }}\right)\;Rx_{2}\left(\phi_{2=0^{\circ }}\right)$	$Tx_{2}\left(\theta_{2=0^{\circ }}\right)\;Rx_{3}\left(\phi_{3=7.5^{\circ }}\right)$
15°	$Tx_{3}\left(\theta_{3=7.5^{\circ }}\right)\;Rx_{3}\left(\phi_{3=7.5^{\circ }}\right)$	$Tx_{2}\left(\theta_{2=0^{\circ }}\right)\;Rx_{5}\left(\phi_{5=15^{\circ }}\right)$

**TABLE 2.** Transmission settings for simulation and phantom experiments

*Transducer Transmit and Receive*	

Transmit Voltage	18 V
Transmit Center Frequency	31.25 MHz
Sampling Rate	62.5 MHz
Transmit Cycles	2
*f* - number	2
Transmit Angles	−7.5, 0, 7.5 degrees
Receive Processing Angles ($Tx_{1}$ and $Tx_{3}$)	−7.5, 0, 7.5 degrees
Receive Processing Angles StaBle ($Tx_{2}$)	−15, −7.5, 0, 7.5, 15 degrees

*Constant Velocity Simulation*	

Window length (*P*)	32 frames
Absolute $PRF_{1}$	6 kHz
Absolute $PRF_{2}$	4, 4.5, 4.8, 5 kHz,
*p/q* ratio	2/3, 3/4, 4/5, 5/6
Nyquist limit StaBle sequence	14.8, 22, 29.4, 36.7 cm/s

*Varying Velocity Simulation*	

Window length (*P*)	16, 32, 64 frames
*p/q* ratio	2/3
Absolute $PRF_{1}$	25 kHz
Absolute $PRF_{2}$	16.67 kHz
Nyquist limit StaBle sequence	61.6 cm/s

*Experimental Phantom*	

Absolute $PRF_{1}$	6 kHz
Absolute $PRF_{2}$	4.5 kHz
p/q ratio	3/4
Nyquist limit Sequential Tx sequence	2.4 cm/s
Nyquist limit Double Tx sequence	7.3 cm/s
Nyquist limit StaBle & Double Tx (3xPRF)	22 cm/s

**TABLE 3.** Transmission settings for in vivo experiment

*In vivo*	

Transmit Center Frequency	31.25 MHz
Sampling Rate	125 MHz
Transmit Voltage	25 V
Transmit Cycles	3
Window length	32 frames
*f* - number	2
Transmit Angles	−7.5, 0, 7.5 degrees
Receive Processing Angles ($Tx_{1}$ and $Tx_{3}$)	−7.5, 0, 7.5 degrees
Receive Processing Angles StaBle ($Tx_{2}$)	−15, −7.5, 0, 7.5, 15 degrees
*p/q* ratio	2/3
Absolute $PRF_{1}$	25 kHz
Absolute $PRF_{2}$	16.67 kHz
Nyquist limit Double transmit sequence	30.8 cm/s
Nyquist limit StaBle sequence	61.6 cm/s
